# Machine learning-based model to predict von Mises stress and chip reduction coefficient developed during dry turning of EN36C steel

**DOI:** 10.1177/00368504251349973

**Published:** 2025-07-21

**Authors:** Vishal Mishra, Nikhil Bharat, Kalyan Chakraborty, Vijay Kumar, Mridusmita Roy Choudhury

**Affiliations:** 1 Centre for Additive Manufacturing, 560142 Chennai Institute of Technology, Chennai, Tamil Nadu, India; 2 Department of Mechanical Engineering, National Institute of Technology Silchar, Cachar, Assam, India; 3 Department of Mechanical Engineering, Graphic Era (Deemed to be University), Dehradun, Uttarakhand, India; 4 Department of Mechanical and Industrial Engineering, Manipal Institute of Technology Bengaluru, Manipal Academy of Higher Education, Manipal, Karnataka, India

**Keywords:** Turning, von Mises stress, Chip reduction coefficient, ANOVA, ANN

## Abstract

This study comprehensively investigates the determination of chip reduction coefficient (CRC) and von Mises stress (VMS) during dry turning of Nickel-Chromium case-hardened steel (EN36C), renowned for its high surface hardness and core toughness. Machining parameters, including cutting speed (36–100 m/min), feed rate (0.49–0.86 mm/rev), and depth of cut (0.67–1.5 mm), were rigorously analyzed using Analysis of Variance (ANOVA) and Artificial Neural Networks. ANOVA identified cutting speed as the most influential factor, accounting for 52.04% of CRC and 35.04% of VMS variations, with feed rate and depth of cut also playing significant roles. ANN modeling achieved a correlation coefficient of 0.97, demonstrating excellent predictive accuracy for parameter optimization. Scanning Electron Microscopy revealed chip morphology, showing continuous chips under optimal conditions of high cutting speed (100 m/min), low feed rate (0.63 mm/rev), and moderate depth of cut (1.0 mm), minimizing stress and enhancing material removal efficiency. Brittle chips were observed at lower speeds (36 m/min) and higher feed rates, emphasizing the critical role of parameter selection. Optimal machining parameters significantly improved surface quality, reduced tool wear, and minimized operational stresses. This research offers a robust framework for machining process optimization, with implications for enhancing industrial efficiency and cost-effectiveness.

## Introduction

The efficiency and reliability of machining operations are critical to modern manufacturing, especially when dealing with hard-to-machine materials. Dry turning, a sustainable alternative to conventional methods, eliminates the need for cutting fluids but increases demands on tool performance and process stability. This study focuses on optimizing dry turning of nickel-chromium case-hardened steel (EN36C), which is known for its exceptional wear resistance and toughness, making it ideal for components such as gears and shafts. However, these very properties also make EN36C challenging to machine, warranting a systematic investigation into its behavior under varying cutting conditions. EN36C is most widely used as an alloy steel for manufacturing gears, shafts, and heavy-duty machine parts, which require high surface hardness and core toughness. The composition of EN36C typically includes 0.12–0.18% carbon, 3.0–3.75% nickel, and 0.80–1.20% chromium, along with small amounts of alloying elements like manganese, silicon, and molybdenum.^1,2^ This steel has the ability to achieve surface hardness with a tough and ductile core, making it suitable for components that require both strength and durability under extreme loading conditions. The surface hardness of EN36C steel was achieved by the case hardening process, which typically involves carburizing. This steel provides high wear resistance on the surface combined with a tough, impact-absorbing core. However, the characteristics that make EN36C steel suitable for such applications also pose significant challenges during machining, particularly turning operations. The increased hardness of the surface layers leads to increased cutting forces, higher tool wear, and elevated temperatures during machining. This makes the turning process more complex, as it requires precise control of parameters to avoid excessive tool wear and maintain the integrity of the workpiece. Additionally, EN36C steel exhibits a work-hardening effect during machining. As the cutting tool penetrates the hardened surface, it induces strain hardening in the material, which further increases the resistance to cutting. This phenomenon not only raises the cutting forces but also leads to more rapid tool wear and increased thermal stress. Combined with the toughness of the core, these factors demand careful optimization of the machining process to ensure efficiency and minimize damage to both the tool and the workpiece. Optimizing the turning process for EN36C steel is therefore crucial to achieving a balance between surface quality, machining efficiency, and tool longevity. Key parameters such as cutting speed, feed rate, depth of cut, and tool material must be carefully selected and balanced to mitigate the challenges posed by the material's hardness and toughness. Due to the complex interactions between these parameters, systematic optimization methods are necessary to identify the most effective combinations. This ensures improved machining outcomes and reduces operational costs by extending tool life and enhancing productivity.

In order to ensure an optimized machining with higher production, researchers used different optimizing techniques to minimize the cutting forces, surface roughness, tool wear, and energy consumption, while maximizing material removal rate and machining efficiency. In the same context, different optimization techniques like Taguchi method,^3–6^ response surface methodology (RSM),^7–10^ Grey Relational Analysis (GRA),^11,12^ metaheuristic analysis (MA),^13,14^ hybrid optimization (like Grey-Fuzzy),^15^ and machine learning (ML)^16–18^ have been employed to optimize machining parameters and enhance performance metrics such as cutting forces, surface quality, tool wear, and material removal rate. Maurya and Niranjan^19^ optimized residual stresses (RS), material removal rate (MRR) and tool wear properties during the computer numerical control (CNC) machining of EN36C steel using response surface methodology (RSM). The minimum RS was 180.528 MPa at cutting speed (V_c_), feed rate (f), and depth of cut (d) of 250 rpm, 0.08 mm/rev and 1.914 mm, respectively. Similarly, Verma et al.^20^ optimized surface roughness of EN36C steel using RSM integrated with Taguchi method. Nagesh and Kumar^21^ optimized the high-speed machining parameters of EN8 steel using the PROMETHEE-II method. The favourable machining parameters were found at V_c_ of 200 m/min, f of 0.08 mm/rev, and d of 0.2 mm, achieving an MRR of 2.887 mm3/s and surface roughness of 0.744 µm. Agarwal et al.^22^ investigated the effect of cutting parameters (V_c_, f and d) on surface roughness and MRR during the CNC turning of 16MnCr5 steel. The parametric optimization was conducted using Taguchi and the Analysis of variance (ANOVA) method. The optimal MRR of 4888 mm^3^/min was found at V_c_, f and d of 200 mm/min, 0.18 mm/rev and 1.5 mm, respectively. In another study, multi-objective optimization was carried out in the dry turning of austenite stainless steel 304 using a Dung Beetle optimizer (meta-heuristic algorithm), a backpropagation neural network, and a Particle Swarm Optimization (PSO) method. It was observed that the surface RS in the cutting direction decreased by 38.47%, while the MRR increased by 91.69%.^23^ Micietova et al.^24^ investigated the residual stresses (RS) induced by dry turning of high-tempered steel using the X-ray diffraction (XRD) technique. It was found that increasing flank wear increases the depth of the cut, inducing compression RS in the material. In another study, the dry-turning of CNC machining was done on annealed and hardened UNIMAX tool steel and its parametric optimization was conducted using Grey Wolf (GW), Multi-Verse (MV) and Ant Lion (AL) metaheuristic algorithms.^25^ Rathod et al.^26^ optimized the hard turning parameters on SS304 steel using grey-based Taguchi and regression methods. It was found that hard-turning operations resulted in a good tool life of approximately 136 min and a production time of around 78 min. The grey-based Taguchi and regression analysis showed the optimal results parameter at V_c_ of 580 m/min, f of 0.13 mm/rev and d of 0.25 mm. Kumar et al.^15^ used PVD-coated (TiAlN) carbide insert in dry hard turning of AISI D2 steel and applied Grey-Fuzzy Hybrid Optimization and Cascade Neural Network Modelling to predict the ool-flank wear, chip morphology, and chip reduction coefficient (CRC). Similarly, in another study, a predictive model using multiple regression analysis (MRA), ANN, and genetic programming (GP) was used to predict the surface roughness of hard machined 40× steel shaft having a hardness of 45HRC. It was found that MRA and GP have lower confidence predictive criteria value than the ANN model.^27^

Although numerous studies have applied statistical and machine learning techniques to optimize machining responses such as surface roughness, material removal rate, and tool wear, limited research has focused on modeling CRC and von Mises stress (VMS) during dry turning of hard-to-machine steels like EN36C. EN36C steel, known for its high surface hardness and core toughness, poses significant challenges during machining due to increased tool wear and thermal loads. While artificial neural networks (ANN) have been employed in some machining applications, their use for predicting CRC and VMS, especially in combination with statistical validation (e.g. ANOVA) and morphological analysis, remains underexplored. Additionally, there is a lack of studies that integrate experimental findings with scanning electron microscopy (SEM) observations to interpret chip formation mechanisms concerning mechanical stresses. This study addresses these gaps by developing an ANN model using the Levenberg–Marquardt algorithm to predict CRC and VMS based on cutting speed, feed rate, and depth of cut. It further employs ANOVA to identify significant influencing parameters and uses SEM to characterize chip morphology under varying conditions. The objectives are to experimentally investigate the influence of machining parameters on CRC and VMS, model and predict these responses using ANN, validate parameter significance using ANOVA, and correlate chip morphology with stress behavior to provide a comprehensive understanding of material response during dry turning of EN36C.

## Materials and methodology

### Materials

In the present work, an EN36C alloy steel round bar with a diameter of 110 mm and a length of 400 mm was selected as a workpiece for the machining. The workpiece comprises a balanced percentage of iron, 0.159% Carbon, 0.386% Manganese, 0.182% Silicon, 0.0164% Phosphorous, 0.820% Chromium, 0.131% Molybdenum, 3.10% Nickel, 0.0182% Aluminium, and 0.0199% Sulphur.^28^ ASBNR25 × 25 M12-A (Make: Tungaloy) tool holder was used to clamp the tool insert. It has a double clamping system with a principal edge angle of 15⁰ and a relief angle of 0⁰. Moreover, the tool has a shank of dimension 25 mm × 25 mm with a square hole to hold the insert. Carbide insert SNMG12040TM T9125 was used for the machining. The insert has a cylindrical hole groove with a cutting-edge length of 12 mm and a 4 mm corner radius.

**Figure 1.**
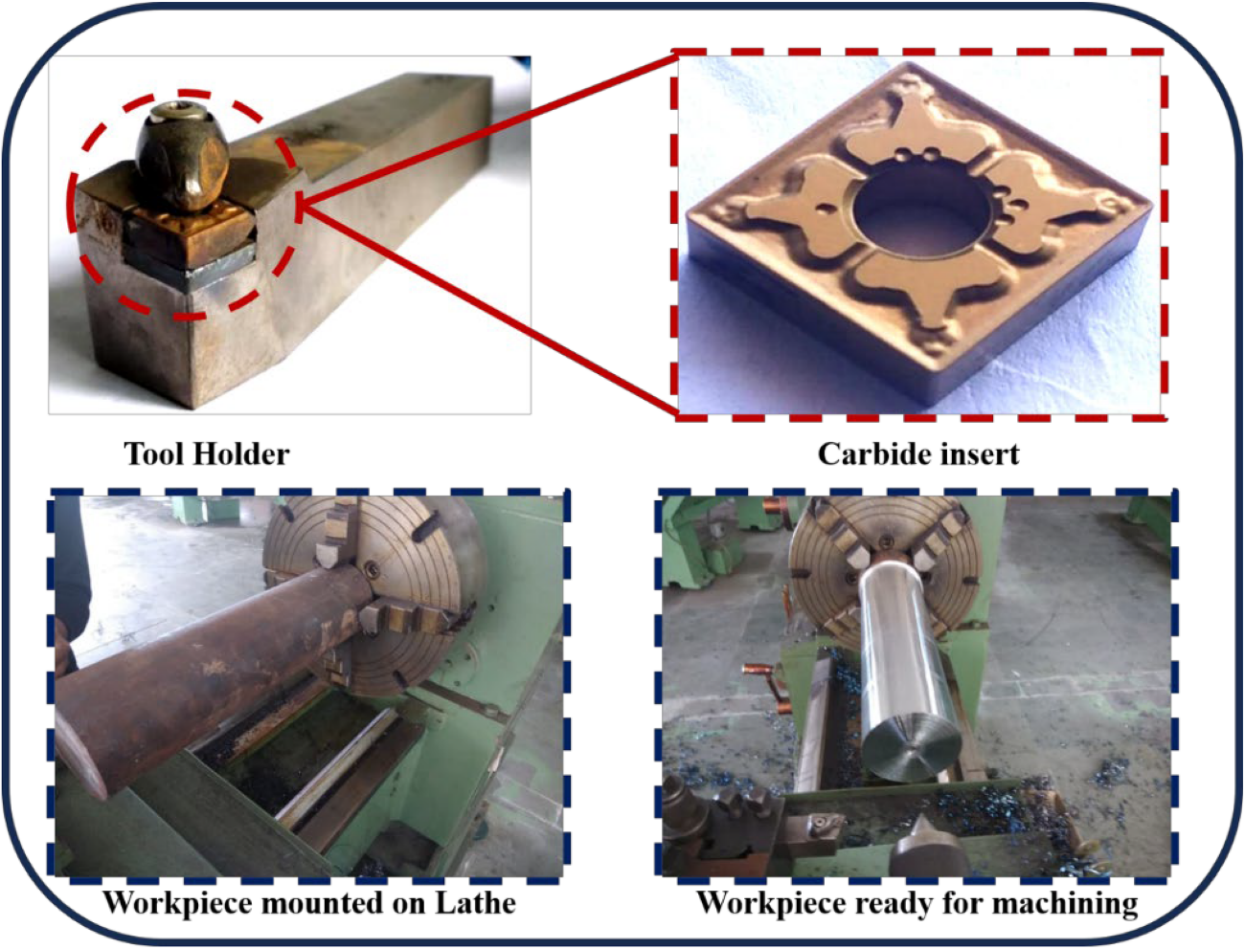
Pictorial view of tool holder, carbide insert and the EN36C steel mounted on GD lathe.^29^

The workpiece was mounted in a four-jaw chuck on a GD-series conventional lathe machine (Make: Champion Engg. Works, India), featuring a maximum spindle speed of 1000 rpm and a motor power of 7.5 HP. The machine is equipped with mechanical feed and thread-cutting capabilities, suitable for medium-duty operations. The dry turning experiments were conducted without any lubrication or coolant, under ambient temperature conditions (approx. 27°C). Each machining trial was conducted for a fixed length of 50 mm with constant engagement to ensure consistency. Tool flank wear was monitored intermittently to ensure tool condition remained within acceptable limits (ISO 3685 standard). The setup ensured rigid clamping and minimal vibration through the use of a dead center support at the tailstock. The physical illustration of the tool and setup is shown in Figure 1.^29^

### Selection of input parameters

The machining process parameters used for the test were determined based on the lathe's cutting speed (*v*), feed rate (*f*), and depth of cut (*d*), calculated using Equation 1.^28^

(1) $$Code(v,f,d)=\frac{\log\;(v,f,d)-\log{\left(v,f,d\right)}_{m}}{\log{\left(v,f,d\right)}_{max}-\log{\left(v,f,d\right)}_{m}}$$

where, *code (v, f, d)* represents the values assigned to different codes corresponding to variation in the cutting speed (*v*), feed (*f*) and depth of cut (doc). *(v, f, d)_m_* indicates the moderate level parameters and *(v, f, d)_max_* indicates the maximum level parameters.

The machining process parameters that are used for the experimentation are shown in Table 1.^29^ Using the specified input process parameters, 3^3^ factorial designs of experiments were conducted, resulting in 27 unique combinations. Five replicates were conducted for each experiment, and the average value was calculated for the analysis. The corresponding CRC and VMS values for each combination are presented in Table 2.^29^

**Table 1.** Input process parameters used for machining.^29^

Factors	Unit	Level 1	Level 2	Level 3
*v*	mm/min	36	60	100
*f*	mm/rev	0.49	0.63	0.86
*doc*	mm	0.67	1	1.5

**Table 2.** Experimental output for the given input parameters.^29^

Exp. Run	*v*	*f*	*doc*	CRC	VMS
1	36	0.49	0.67	1.78	2359.19
2	60	0.49	0.67	1.32	2067.96
3	100	0.49	0.67	1.04	1468.40
4	36	0.63	0.67	1.39	2134.56
5	60	0.63	0.67	1.19	1907.80
6	100	0.63	0.67	1.01	1032.23
7	36	0.86	0.67	1.46	2191.49
8	60	0.86	0.67	1.43	2167.56
9	100	0.86	0.67	1.33	2080.24
10	36	0.49	1	1.92	2412.35
11	60	0.49	1	1.15	1829.80
12	100	0.49	1	1.11	1746.90
13	36	0.63	1	1.71	2326.72
14	60	0.63	1	1.08	1639.76
15	100	0.63	1	1.07	1605.15
16	36	0.86	1	1.23	1963.79
17	60	0.86	1	1.01	1099.85
18	100	0.86	1	1.06	1552.18
19	36	0.49	1.5	1.46	2191.17
20	60	0.49	1.5	1.45	2183.32
21	100	0.49	1.5	1.13	1797.76
22	36	0.63	1.5	1.27	2013.03
23	60	0.63	1.5	1.03	1351.82
24	100	0.63	1.5	1.04	1447.55
25	36	0.86	1.5	1.59	2268.70
26	60	0.86	1.5	1.33	2078.24
27	100	0.86	1.5	1.31	2064.43

The cutting speed (36–100 m/min), feed rate (0.49–0.86 mm/rev), and depth of cut (0.67–1.5 mm) were selected based on a combination of machine tool limitations, tool manufacturer recommendations, and ranges reported in prior literature for dry turning of hard or case-hardened steels such as EN36C. The lower limits (36 m/min, 0.49 mm/rev, and 0.67 mm) reflect conservative settings recommended for minimizing tool wear during dry machining, particularly when dealing with high surface hardness. The upper limits (100 m/min, 0.86 mm/rev, and 1.5 mm) align with prior studies that explored optimal cutting conditions for EN36C steel using CNC lathes.^19,20^ Additionally, the selected ranges are consistent with tool manufacturer's guidance (Tungaloy - SNMG12040TM T9125) for dry machining of hardened steels. These parameters also ensured safe operation within the spindle power (7.5 HP) and structural rigidity limits of the lathe used in this study, allowing meaningful analysis of their influence on CRC and VMS .

### Calculation of CRC and VMS

The CRC and VMS are calculated using below equations given below (2–6).^29^

(2) $$Uncut\;Chip\;Thickness\;(t_{1})=f\times sin\varphi$$

where, f is the feed rate (mm/rev) and φ is the principle cutting edge angle (degrees)

(3) $$Cut\;chip\;thickness\;(t_{2})=\frac{W}{\rho wl}$$

where, *W, ρ, l* and *w* were represented as chip weight (g), the density of the steel (0.008 g/mm^3^), the chip length (mm) and the chip width (mm), respectively.

(4) $$width\;of\;a\;chip\;(w)=\frac{d}{cos\varphi }$$

where, d and φ are the doc (mm) and principle cutting edge angle (degrees)

(5) $$\mathrm{CRC}=\frac{t{}_{2}}{t{}_{1}}$$

(6) $$VMS=1.74\times K\times (\ln\;CRC{)}^{n}\;$$

where, K and n are the strength coefficient (MPa) and strain hardening exponent, respectively. The K and n values are derived from the true stress-strain curve of EN36C steel and taken as 1495 MPa and 0.178, respectively.

### Analysis of variance analysis (ANOVA)

Analysis of variance (ANOVA) is a statistical method that compares the means of the groups to determine the significant differences between them. Its core principle is to split the total variability that is observed in the data into different components attributed from different sources, like variability between the groups and variability within the groups. The signal-to-noise (S/N) ratio is used to quantify the variation in the process with respect to the desired outcome. For the smaller-is-better quality characteristic, where the goal is to minimize the response, the S/N ratio formula is given by Equation 7. The S/N ratio represents the robustness of the system.

(7) $$\frac{S}{N}=-10\times log_{10}\left(\frac{1}{n}\sum_{i=1}^{n}\;y_{i}^{2}\right)$$

where, n is the of observations (samples) in a trial, and $\left(\frac{1}{n}\sum_{i=1}^{n}\;y_{i}^{2}\right)$ is the mean of the squared values of the response, quantifying the undesirable characteristic's variability and magnitude.

The above equation focuses on minimizing the variance and the magnitude of the output. By taking the negative logarithm, the S/N ratio converts variability into a logarithmic scale, ensuring that smaller values of y_i_ (closer to the ideal target of zero) result in higher S/N ratios, indicating better performance. This approach is particularly useful in manufacturing and quality control to achieve a stable and consistent process.

### Levenberg-Marquardt neural network algorithm

The Levenberg-Marquardt Neural Network (LM-NN) algorithm is an advanced optimization technique that is used to train feedforward neural networks specifically for regression and function approximation tasks. LM-NN is a combination of gradient descent and the Gauss-Newton method that offers a balance between convergence speed and numerical stability. This algorithm minimizes the error function by iteratively updating the weights and biases of the neural network. Additionally, this algorithm follows the update rule to adjust the parameters given by Equation 13.

(13) $$\Delta w=-\;[J^{T}J+\mu I{]}^{-1}J^{T}e$$

where, J is the Jacobian matrix of the network errors with respect to weights, e is the error vector, μ is a damping parameter, and I is the identity matrix. When μ is small, the algorithm approximates the Gauss-Newton method for faster convergence, whereas a larger μ shifts it towards gradient descent for improved stability in non-linear regions. The algorithm adaptively adjusts μ during training based on error reduction. Its efficiency and ability to handle complex, non-linear problems make LM-NN a preferred choice for small to medium-sized datasets where computational cost is manageable.

### Chip and tool wear morphology

Chip morphology was analyzed using Field Emission Scanning Electron Microscopy (FE-SEM) (Make: Nova, Model: NANOSEM 450) with a resolution of 1 nm at 30 kV, providing high magnification imaging of chip surfaces and cross-sections. Before observation, chip samples were cleaned in acetone using an ultrasonic bath for 10 min to remove debris and ensure a clear surface. For cross-sectional analysis, selected chip samples were cold-mounted in epoxy resin, ground with SiC papers (up to 1200 grit), and polished using diamond paste down to a 1 µm finish. Final cleaning was done with ethanol, followed by air-drying. These preparation steps ensured minimal deformation or contamination that could affect morphology analysis. All SEM imaging was conducted under high vacuum with consistent working distance and acceleration voltage to maintain image fidelity and comparability across samples. The preparation process was carefully controlled to avoid introducing artifacts that could alter chip morphology interpretation.

## Results and discussion

### Analysis of variance (ANOVA) analysis

#### ANOVA for CRC

The ANOVA data shown in Table 3 provide a detailed analysis of the factors influencing the CRC during the turning of EN36C steel. From the results, it is evident that cutting speed has the most significant effect on CRC, contributing 52.04% of the total variation, with a high F-value of 37.36 and a p-value of 0.000, indicating a strong statistical significance. The feed rate also significantly influences CRC, contributing 9.35% to the variation, with an F-value of 6.72 and a p-value of 0.019, confirming its importance. However, the depth of cut shows minimal influence, contributing only 2.17% to the variation, with an F-value of 1.56 and a p-value of 0.267, suggesting it is not statistically significant.

**Table 3.** Analysis of variance for SN ratios for CRC.

Source	DF	Seq SS	Adj SS	Adj MS	F	P	%Contribution
Cutting Speed	2	34.675	34.675	17.3375	37.36	0.000	52.04
Feed	2	6.234	6.234	3.1171	6.72	0.019	9.35
doc	2	1.452	1.452	0.7261	1.56	0.267	2.17
Cutting Speed × Feed	4	5.501	5.501	1.3752	2.96	0.089	8.25
Speed × doc	4	5.180	5.180	1.2950	2.79	0.101	7.77
Feed × doc	4	9.865	9.865	2.4662	5.31	0.022	14.80
Residual Error	8	3.713	3.713	0.4641			5.57
Total	26	66.619					
R-Sq	94.43%						
R-Sq(adj)	81.89%						

Interactions between factors also play a role. The interaction between speed and feed contributes 8.25% of the variation, while the interaction between speed and depth of cut contributes 7.77%, though neither is statistically significant, with p-values of 0.089 and 0.101, respectively. Conversely, the interaction between feed and depth of cut is statistically significant, contributing 14.8% of the variation with an F-value of 5.31 and a p-value of 0.022.

The residual error accounts for 5.57% of the variation, representing unexplained variability. The model has an R-squared value of 94.43%, indicating that it explains a substantial portion of the total variation in CRC. However, the adjusted R-squared value of 81.89% suggests a slight reduction in explanatory power when accounting for the number of predictors in the model.

The response table shown in Table 4 for signal-to-noise ratios (SNR) under the “smaller is better” criterion indicates that the cutting speed has the most significant influence on the CRC. As the speed increases from Level 1 to Level 3, the SNR improves substantially, with values moving from −3.6342 to −0.9596. This highlights the dominant effect of speed on the response. Feed rate also affects the SNR, with Level 2 (−1.4330) providing the best performance compared to Levels 1 and 3. However, its impact is less pronounced than that of speed. The depth of cut has the least influence, showing only slight variation across levels, with Level 2 (−1.7802) being slightly better than the other levels. The delta values, which represent the difference between the highest and lowest SNR for each factor, confirm the ranking of their influence. Cutting speed has the highest delta (2.6746), followed by feed rate (1.1475), and depth of cut (0.5637). The ranking of parameters is thus Cutting Speed > Feed > Depth of Cut.

**Table 4.** Response table for SN ratios for CRC.

Smaller is better			
Level	Cutting Speed	Feed	doc
1	−3.6342	−2.5805	−2.3439
2	−1.6533	−1.4330	−1.7802
3	−0.9596	−2.2336	−2.1231
Delta	2.6746	1.1475	0.5637
Rank	1	2	3

**Figure 2.**
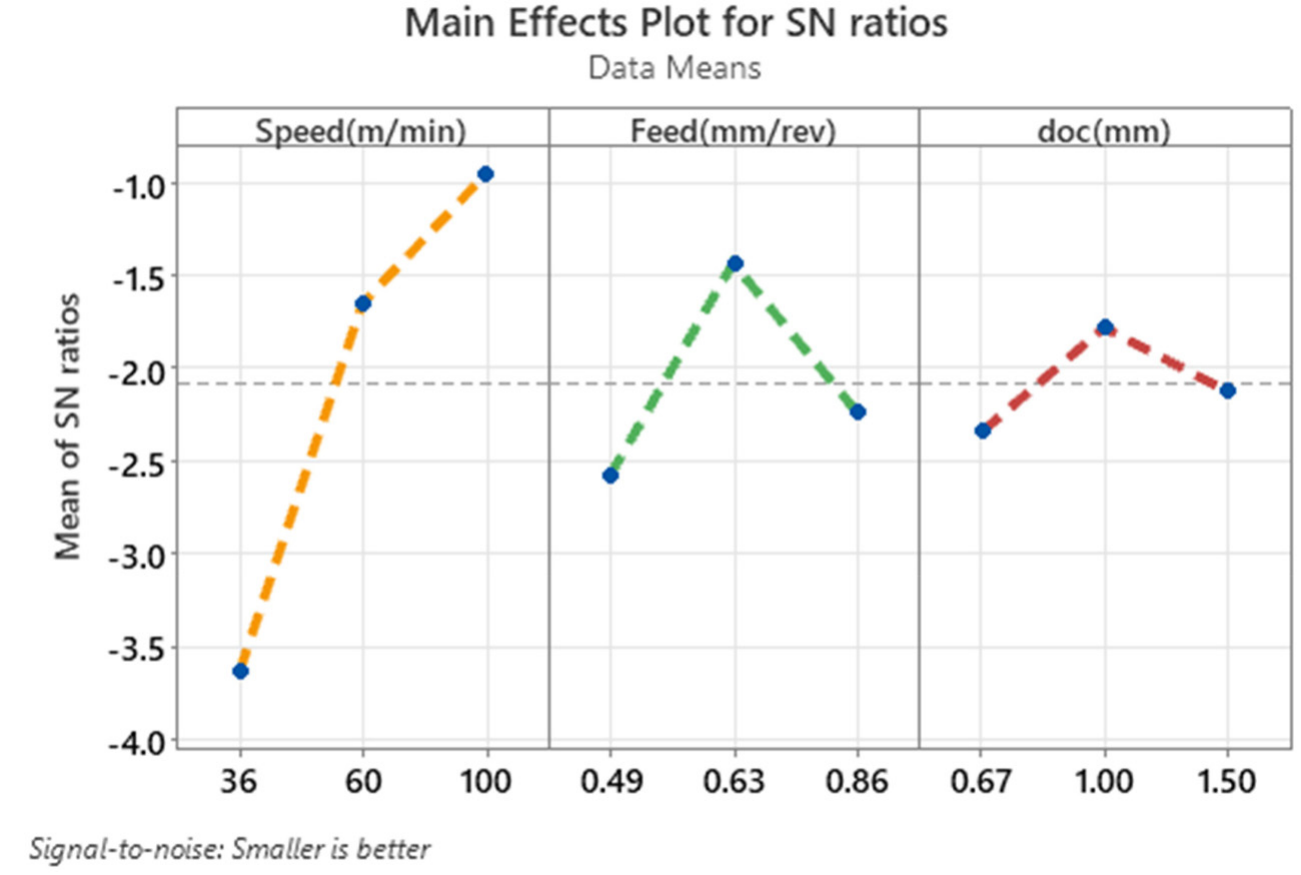
Main effects plot for SN ratios for CRC.

The main effects plot for signal-to-noise (S/N) ratios under the “smaller is better” criterion shown in Figure 2 provides a visual representation of the influence of cutting parameters—cutting speed, feed, and depth of cut—on the CRC. The plot indicates a significant improvement in performance as cutting speed increases, with the mean S/N ratio rising sharply from −3.6 at 36 m/min to −0.95 at 100 m/min. This trend demonstrates that higher speeds result in a smaller CRC, confirming the dominant influence of speed on the response. The feed rate shows a peak in the S/N ratio at 0.63 mm/rev, indicating optimal performance at this level. However, performance declines when the feed is either increased or decreased from this value, suggesting that feed rate has a moderate impact on CRC compared to speed. For the depth of cut, the S/N ratio is highest at 1.0 mm, showing a slight improvement in performance at this level. However, the changes in S/N ratio across the depth of cut levels are minimal, highlighting its relatively low influence on CRC compared to speed and feed. The plot confirms that cutting speed has the most significant impact on CRC, followed by feed rate, while the depth of cut plays a minor role in influencing the response.

**Figure 3.**
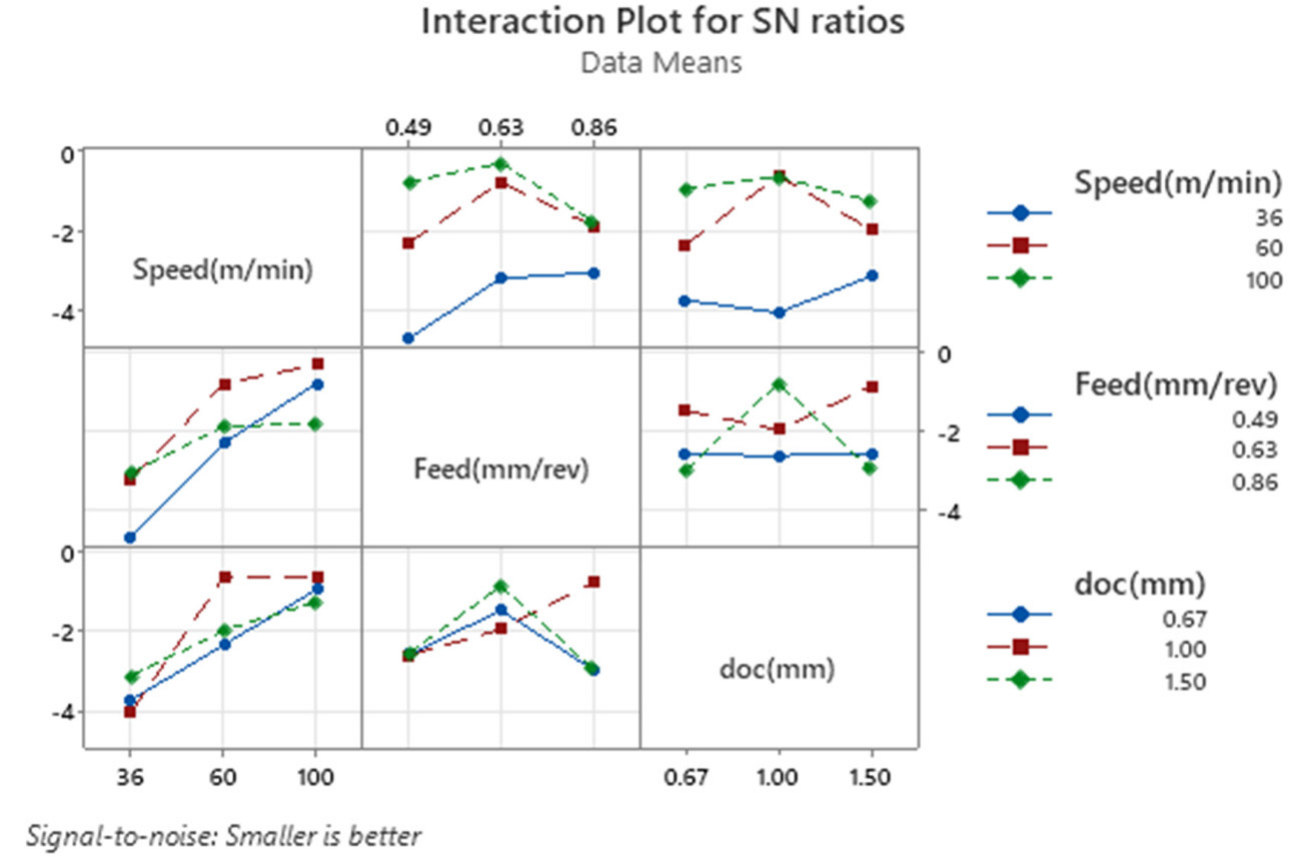
Interaction plot for SN ratios for CRC.

The interaction plot for signal-to-noise (S/N) ratios shown in Figure 3 provides a detailed visualization of the interactions between cutting speed, feed, and depth of cut (doc) on the CRC. Each subplot shows how two factors interact while the third factor is held constant, allowing a deeper understanding of their combined effects. The plot reveals that cutting speed significantly influences the S/N ratio, and its interaction with feed and depth of cut varies. For instance, at higher speeds (100 m/min), the S/N ratio is consistently better across all levels of feed and depth of cut, suggesting a dominant and positive effect of high speeds. However, at lower speeds (36 m/min), the S/N ratio tends to worsen, and the interactions between feed and doc show greater variability. For feed, its interaction with speed is more prominent than with depth of cut. At a moderate feed rate (0.63 mm/rev), the S/N ratio is optimized across different speed levels, indicating this feed setting is generally favorable. When feed is either decreased (0.49 mm/rev) or increased (0.86 mm/rev), the S/N ratio declines, especially at lower speeds. The depth of cut has a relatively weaker effect on the S/N ratio, as evident from its interaction patterns. The plots suggest that the S/N ratio is slightly better at a doc of 1.0 mm for most combinations of speed and feed. However, changes in doc do not lead to significant variation, reinforcing its limited influence compared to speed and feed. The interaction plot confirms that cutting speed has the most significant effect on CRC, with its interactions with feed and depth of cut influencing the outcome to varying degrees. Feed exhibits moderate interactions, while depth of cut shows only minor effects.

**Figure 4.**
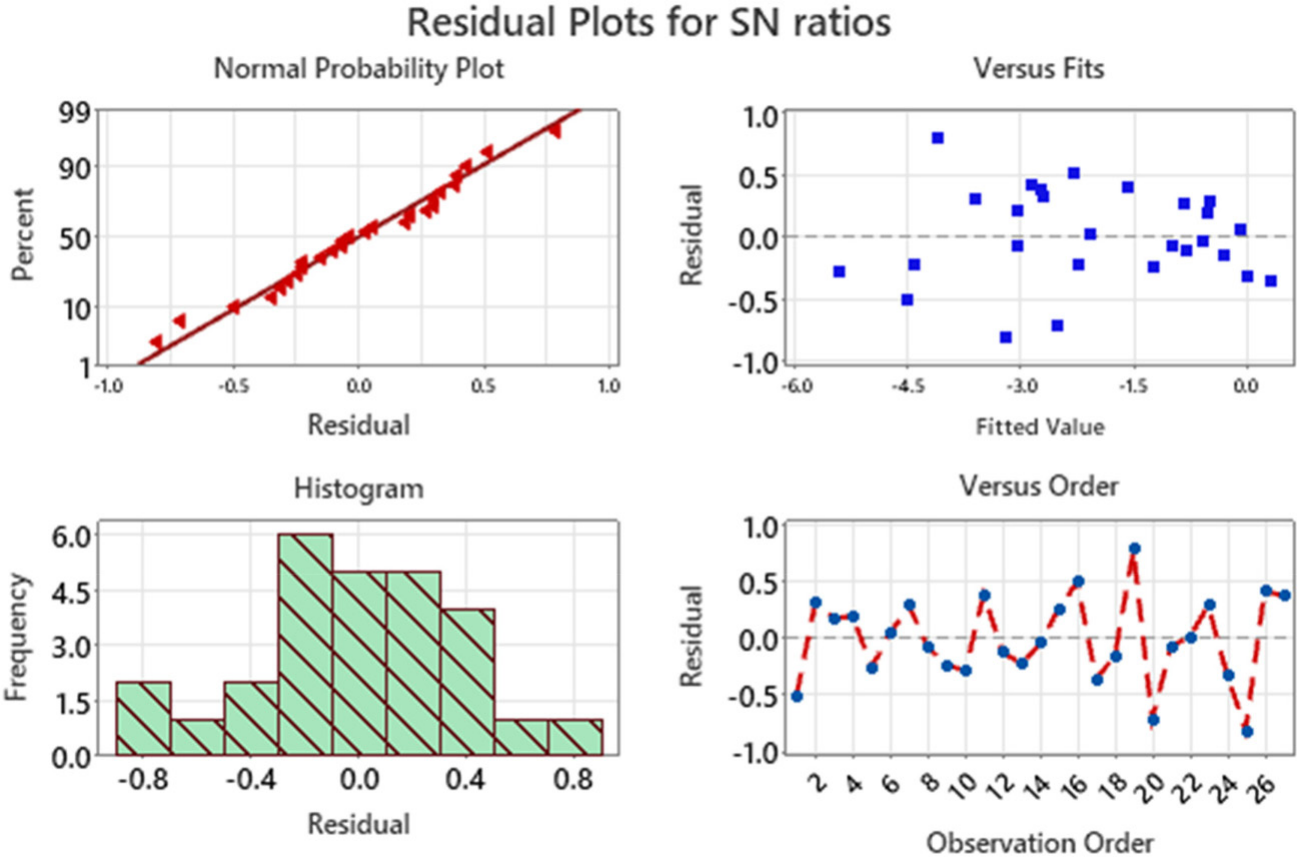
Residual plot for SN ratios for CRC.

The residual plots for the signal-to-noise (S/N) ratios shown in Figure 4 provide a comprehensive assessment of the model's assumptions and its suitability for explaining the variation in the data. Each plot serves a specific purpose in verifying key assumptions, including normality, independence, and constant variance.

The Normal Probability Plot shows the relationship between the residuals and their expected values under a normal distribution. Most of the residual points lie close to the reference line, indicating that the residuals follow a normal distribution. While there are slight deviations at the tails, these are not significant enough to challenge the assumption of normality. This suggests that the statistical model's estimates are likely unbiased and reliable. The Residuals vs. Fits Plot examines the residuals against the predicted values to check for patterns that might indicate issues with the model, such as heteroscedasticity (non-constant variance) or systematic error. In this case, the residuals are randomly scattered around zero, with no discernible pattern or structure. This randomness confirms that the variance of the residuals remains constant across different levels of the fitted values and that the model does not exhibit systematic bias. The Histogram provides a visual representation of the distribution of residuals. The histogram is centered around zero and exhibits a roughly symmetric bell-shaped curve, which supports the assumption of normality. Although the distribution is not perfectly smooth, it does not show any extreme skewness or multimodality, indicating that the residuals are well-behaved and consistent with the model's assumptions. The residuals vs. observation order plot shows the residuals plotted in the order in which the data were collected. The residuals fluctuate randomly around zero without showing any trend, cyclical pattern, or clustering. This randomness implies that the residuals are independent of one another, and there is no time-related or sequential bias affecting the results.

#### ANOVA for VMS

The ANOVA table for the signal-to-noise (S/N) ratios ofVMS presented in Table 5 evaluates the contributions of various factors and their interactions—cutting speed, feed, and depth of cut (doc)—to the observed variation in stress. The analysis provides insight into which factors and interactions significantly affect VMS and how well the model explains the variation. The factor cutting speed has the largest contribution (35.04%) to the variation in S/N ratios, with a high F-value of 16.99 and a very low p-value (0.001), indicating that it is highly significant. This highlights the dominant role of cutting speed in influencing VMS, making it the most critical parameter in the analysis. The feed rate contributes 10.95% to the variation, with an F-value of 5.31 and a p-value of 0.034, which is below the significance threshold. This indicates that feed is also an influential factor, though its effect is less pronounced than cutting speed. The depth of cut (doc), on the other hand, contributes only 2.67% to the variation. Its F-value of 1.30 and a p-value of 0.325 suggest that it is statistically insignificant. This implies that depth of cut has minimal influence on VMS compared to speed and feed. Among the interactions, speed × feed has a moderate contribution (9.84%), with an F-value of 2.39 and a p-value of 0.137, indicating that it is not statistically significant but shows some influence. The interaction speed × depth of cut has a slightly higher contribution (13.03%), with an F-value of 3.16 and a p-value of 0.078, suggesting a marginal effect. The interaction feed × depth of cut contributes 20.18% to the variation, making it the most significant interaction term. Its F-value of 4.89 and a p-value of 0.027 indicate that this interaction is statistically significant, demonstrating a notable combined effect of feed and depth of cut on VMS. The residual error, which represents unexplained variation, accounts for 8.25% of the total variation, showing that most of the variation is captured by the model. The model's goodness-of-fit is reflected by the R-Sq value of 91.75%, indicating that the model explains 91.75% of the total variation in the data. However, the adjusted R-Sq value of 73.19% suggests that the model may include terms with limited significance, and further refinement might improve its predictive capability.

**Table 5.** Analysis of variance for SN ratios for VMS.

Source	DF	Seq SS	Adj SS	Adj MS	F	P	%Contribution
Cutting Speed	2	34.636	34.636	17.318	16.99	0.001	35.04
Feed	2	10.824	10.824	5.412	5.31	0.034	10.95
doc	2	2.647	2.647	1.323	1.30	0.325	2.67
Cutting Speed × Feed	4	9.733	9.733	2.433	2.39	0.137	9.84
Speed × doc	4	12.882	12.882	3.221	3.16	0.078	13.03
Feed × doc	4	19.953	19.953	4.988	4.89	0.027	20.18
Residual Error	8	8.153	8.153	1.019			8.25
Total	26	98.828					
R-Sq	91.75%						
R-Sq(adj)	73.19%						

The response table for signal-to-noise (S/N) ratios presented in Table 6 provides information about the effects of different process parameters—cutting speed, feed rate, and depth of cut—on VMS during machining. The objective here is to minimize VMS, as the goal is to achieve smaller values for the S/N ratio, which is associated with a more stable and desirable process.

**Table 6.** Response table for signal to noise ratios for VMS.

Smaller is better			
Level	Cutting speed	Feed	doc
1	−66.86	−65.95	−65.50
2	−64.97	−64.46	−64.89
3	−64.15	−65.57	−65.60
Delta	2.71	1.49	0.71
Rank	1	2	3

Looking at the data, cutting speed (Speed in m/min) shows the largest variation in the S/N ratio. At Level 1, the S/N ratio is −66.86, at Level 2 it is −64.97, and at Level 3 it is −64.15. The delta for cutting speed is 2.71, indicating that as cutting speed increases, the S/N ratio becomes less negative, implying that higher cutting speeds lead to better outcomes in terms of lower VMS. This means cutting speed has the most significant impact on reducing the stress, making it the most influential factor in this study, ranked number 1. Feed rate (Feed in mm/rev) also affects the S/N ratio but to a lesser extent. The S/N ratios at different feed levels are −65.95 at Level 1, −64.46 at Level 2, and −65.57 at Level 3. The delta for feed is 1.49, which is smaller than that for cutting speed. This shows that increasing the feed rate tends to improve the process (i.e. reduce VMS), particularly at Level 2. Feed rate ranks second in its effect on the process, meaning it has a notable but smaller influence compared to cutting speed. Depth of cut (doc in mm) has the least effect on the S/N ratio. At Level 1, the S/N ratio is −65.50, at Level 2 it is −64.89, and at Level 3 it is −65.60. The delta for depth of cut is 0.71, which is the smallest among the three factors. This suggests that depth of cut does not significantly impact the VMS as much as cutting speed and feed rate do. Consequently, depth of cut is ranked third, indicating it has the smallest effect on the outcome. In conclusion, cutting speed is the most critical factor in minimizing VMS, followed by feed rate, with depth of cut having the least influence on the process. The rankings reflect the relative importance of each factor in controlling the stress during the turning process.

**Figure 5.**
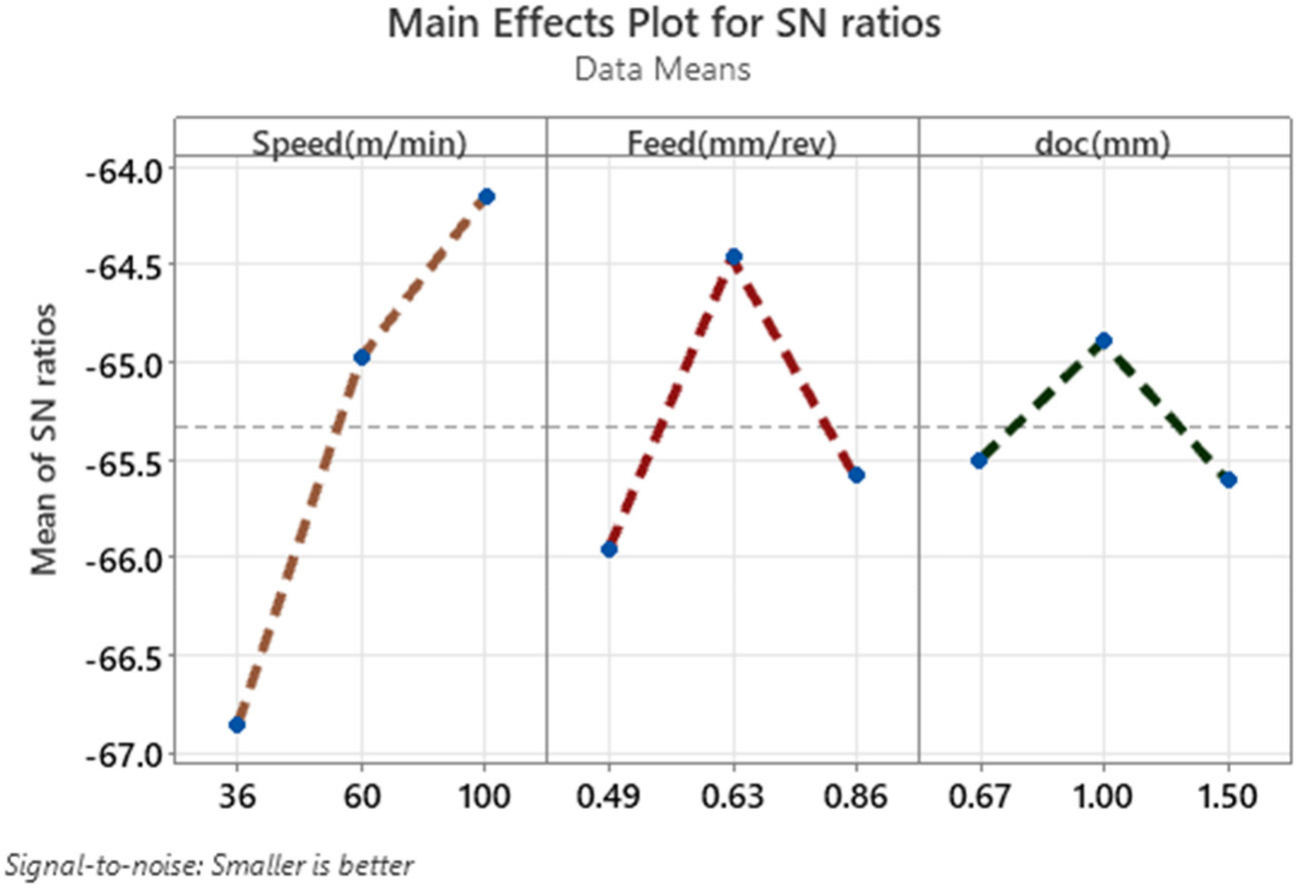
Main effect plot for SN ratio for VMS.

The “Main Effects Plot for S/N Ratios” shown in Figure 5, graphically illustrates the influence of cutting speed, feed rate, and depth of cut on the signal-to-noise (S/N) ratio for VMS during the machining process. The analysis follows the “smaller is better” criterion, meaning lower S/N ratios indicate improved performance by minimizing stress. In the first plot, representing cutting speed (Speed in m/min), there is a noticeable upward trend in the mean of the S/N ratios as the speed increases from 36 to 100 m/min. At the lowest speed (36 m/min), the S/N ratio is approximately −67.0, which is the least desirable. As speed increases to 100 m/min, the S/N ratio improves to around −64.0. This indicates that higher cutting speeds result in lower VMS, making speed the most influential factor in this study. The second plot shows the effect of feed rate (Feed in mm/rev). Here, the S/N ratio follows a parabolic trend, peaking at 0.63 mm/rev. At the lowest and highest feed rates (0.49 and 0.86 mm/rev), the S/N ratios are less favorable, around −65.9 and −65.6, respectively. The optimal feed rate for minimizing stress is at 0.63 mm/rev, where the S/N ratio reaches approximately −64.5. In the third plot, the depth of cut (doc in mm) exhibits a smaller effect on the S/N ratio. The S/N ratio improves as the depth of cut increases from 0.67 mm to 1.0 mm, reaching a peak value of about −65.5. However, as the depth of cut increases further to 1.5 mm, the S/N ratio deteriorates slightly to −65.6. This suggests that while depth of cut has a marginal influence, the best performance occurs at 1.0 mm. Overall, the plot highlights that cutting speed has the most substantial impact on VMS, with higher speeds leading to better outcomes. Feed rate is the second most influential factor, showing an optimal point at 0.63 mm/rev. Depth of cut has the least influence, with a slight improvement at 1.0 mm. These trends align with the earlier findings, where cutting speed ranked as the most critical factor, followed by feed rate and depth of cut.

**Figure 6.**
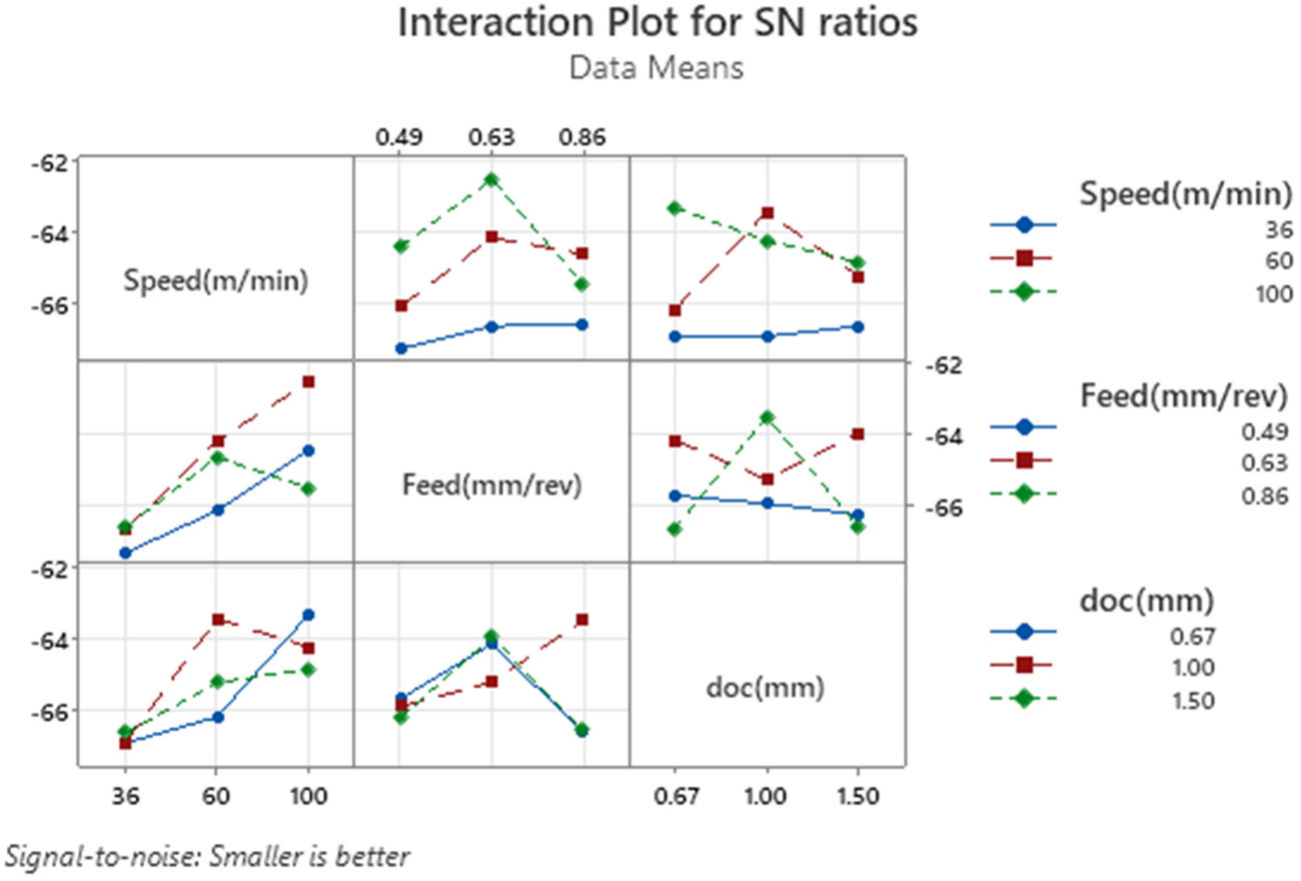
Interaction plot for SN ratio for VMS.

The “Interaction Plot for S/N Ratios” shown in Figure 6 provides an insightful representation of how the machining parameters—cutting speed, feed rate, and depth of cut (doc) interact and influence the signal-to-noise (S/N) ratios for VMS, adhering to the “smaller is better” criterion. The interaction between cutting speed and feed rate illustrates a significant variation in S/N ratios. At the lowest speed of 36 m/min, the S/N ratio remains relatively stable across all feed rates, whereas at higher speeds of 60 and 100 m/min, the S/N ratio improves significantly, particularly at a feed rate of 0.63 mm/rev. This pattern indicates that higher speeds combined with moderate feed rates effectively reduce stress levels, as evident from the non-parallel lines reflecting strong interaction. Similarly, the interaction between speed and depth of cut displays a noticeable trend. At lower speeds, the S/N ratio improves steadily with an increase in depth of cut. However, at higher speeds, the depth of cut of 1.0 mm yields the best S/N ratio, with a slight increase in stress levels at 1.5 mm. This suggests that higher speeds require a specific depth of cut, particularly 1.0 mm, to minimize stress effectively. The crossing lines in this interaction highlight the interdependence of these two parameters. The relationship between feed rate and depth of cut is also crucial, showing an inverted parabolic behavior. Across all feed rates, the depth of cut of 1.0 mm consistently provides the lowest stress levels, while higher feed rates, such as 0.86 mm/rev, paired with a depth of cut of 1.5 mm, result in slightly elevated stress. This demonstrates that depth of cut plays a critical role in reducing stress, especially when combined with moderate feed rates. Overall, the plot emphasizes that cutting speed has the most significant impact on VMS, especially when optimized with the appropriate feed rate and depth of cut. The combination of a high cutting speed (100 m/min), a moderate feed rate (0.63 mm/rev), and a depth of cut of 1.0 mm emerges as the most favorable condition for minimizing stress. The crossing lines across all subplots underscore the importance of understanding the interplay among these machining parameters to achieve optimal results.

**Figure 7.**
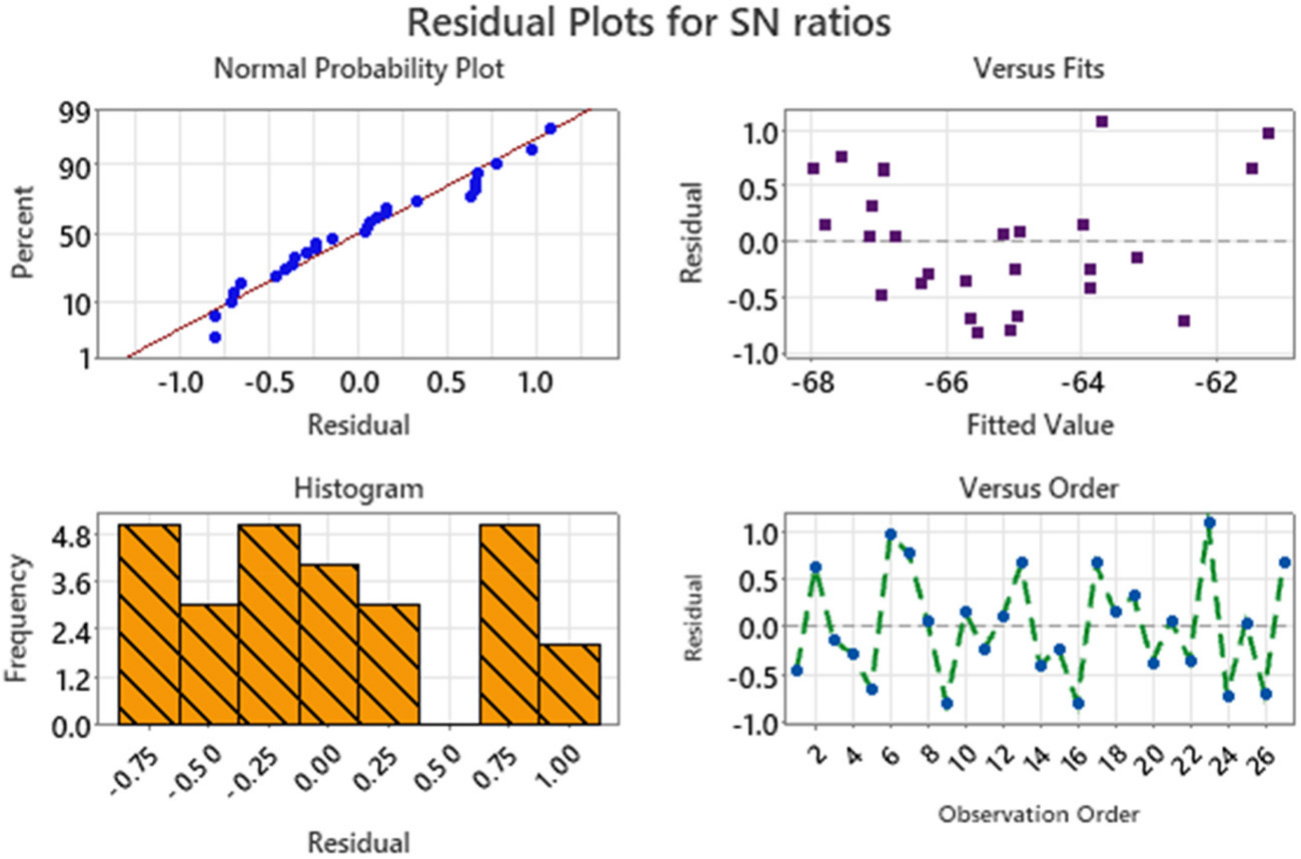
Residual plots for SN ratio for VMS.

The residual plots for signal-to-noise (S/N) ratios shown in Figure 7 provide an in-depth assessment of the residuals to validate the model's assumptions. The normal probability plot demonstrates that the residuals closely follow the diagonal reference line, indicating they are approximately normally distributed, which validates the assumption of normality. The residuals versus fits plot reveals a random distribution of residuals around the zero line, confirming the absence of any systematic patterns or trends. This suggests that the residuals are independent and exhibit constant variance, which is crucial for the reliability of the model. The histogram of residuals shows a relatively balanced distribution around zero, further supporting the assumption of normality. Lastly, the residuals versus observation order plot indicates a random and scattered pattern over the observation sequence, reinforcing the independence of residuals. Together, these plots confirm that the model's assumptions are met, ensuring the robustness and reliability of the analysis.

#### Regression analysis for CRC and VMS

The regression equation for CRC, shown in Equation 14 indicates the effect of cutting speed, feed rate, and depth of cut (DOC) on the chip thickness reduction. Increasing cutting speed reduces CRC slightly, as higher speeds lead to thinner chips. Higher feed rates significantly decrease CRC, as thicker chips form with less reduction. Similarly, increasing DOC lowers CRC as more material is removed per pass, reducing chip compression.

(14) CRC=1.807−(0.00605×Speed)−(0.123×Feed)−(0.037×doc)

The regression equation for VMS shown in Equation 15 models the stress experienced by the material during cutting. Increasing cutting speed decreases VMS, as thermal softening reduces material strength. Higher feed rates increase VMS due to greater cutting forces while increasing DOC also raises VMS by concentrating stress at the cutting edge. These factors collectively influence material deformation and failure during machining.

(15) VMS=2453−(8.33×Speed)−(64×Feed)−(20×doc)

Table 7 presents the confirmatory test results for CRCand VMS under machining conditions of a cutting speed of 100 m/min, a feed rate of 0.63 mm/rev, and depths of cut of 0.67 mm for CRC and 1 mm for VMS. The actual and predicted values show errors of 6.71% for CRC (actual: 1.0056, predicted: 1.078) and 10.72% for VMS (actual: 1605.1, predicted: 1797.9), both of which fall within the acceptable range, confirming the validity of the ANOVA-based prediction model.

**Table 7.** Confirmatory test analysis for CRC and VMS.

Output	Process Parameters					
	speed(m/min.)	feed(mm/rev.)	doc(mm)	Actual	Predicted	Error%
CRC	100	0.63	0.67	1.0056	1.078	6.71
VMS	100	0.63	1	1605.1	1797.9	10.72

### Levenberg-Marquardt neural network algorithm analysis

The Levenberg-Marquardt (LM) algorithm is an optimization technique that combines the advantages of gradient descent and the Gauss-Newton method, providing fast and effective solutions for minimizing error functions in non-linear systems. In the context of turning EN36C steel, LM is used to adjust parameters like cutting speed, feed rate, and depth of cut to minimize the difference between actual and predicted values of the CRC and VMS, thereby improving the accuracy of the model. This algorithm is particularly suitable for small to medium-sized datasets due to its relatively high computational cost. Moreover, the dynamic adjustment of the learning rate makes it robust and less sensitive to initial parameter settings.^30,31^ Therefore, LM is considered an effective method for achieving rapid convergence and high performance when modeling CRC and VMS during the turning of EN36C steel.

Additionally, to validate the robustness of the ANN model, the dataset was randomly divided into three sets: training (70%), validation (15%), and testing (15%). The model's performance was assessed based on the correlation coefficient (R-value), mean squared error (MSE), and error distribution across all datasets. High R-values (above 0.94) across training, validation, and testing stages indicated strong predictive capability. However, several assumptions and limitations should be acknowledged. The model assumes that the relationships between inputs and outputs are consistent across the design space and that there are no significant unmodeled external influences. Given the limited dataset size (27 experimental runs), the ANN model may be prone to overfitting if excessively complex architectures are used. To mitigate this, early stopping based on validation performance was implemented, as seen in the best performance occurring at epoch 2. Additionally, the model was not exposed to extrapolative data outside the training range, so its predictive accuracy is only guaranteed within the tested parameter space.

**Figure 8.**
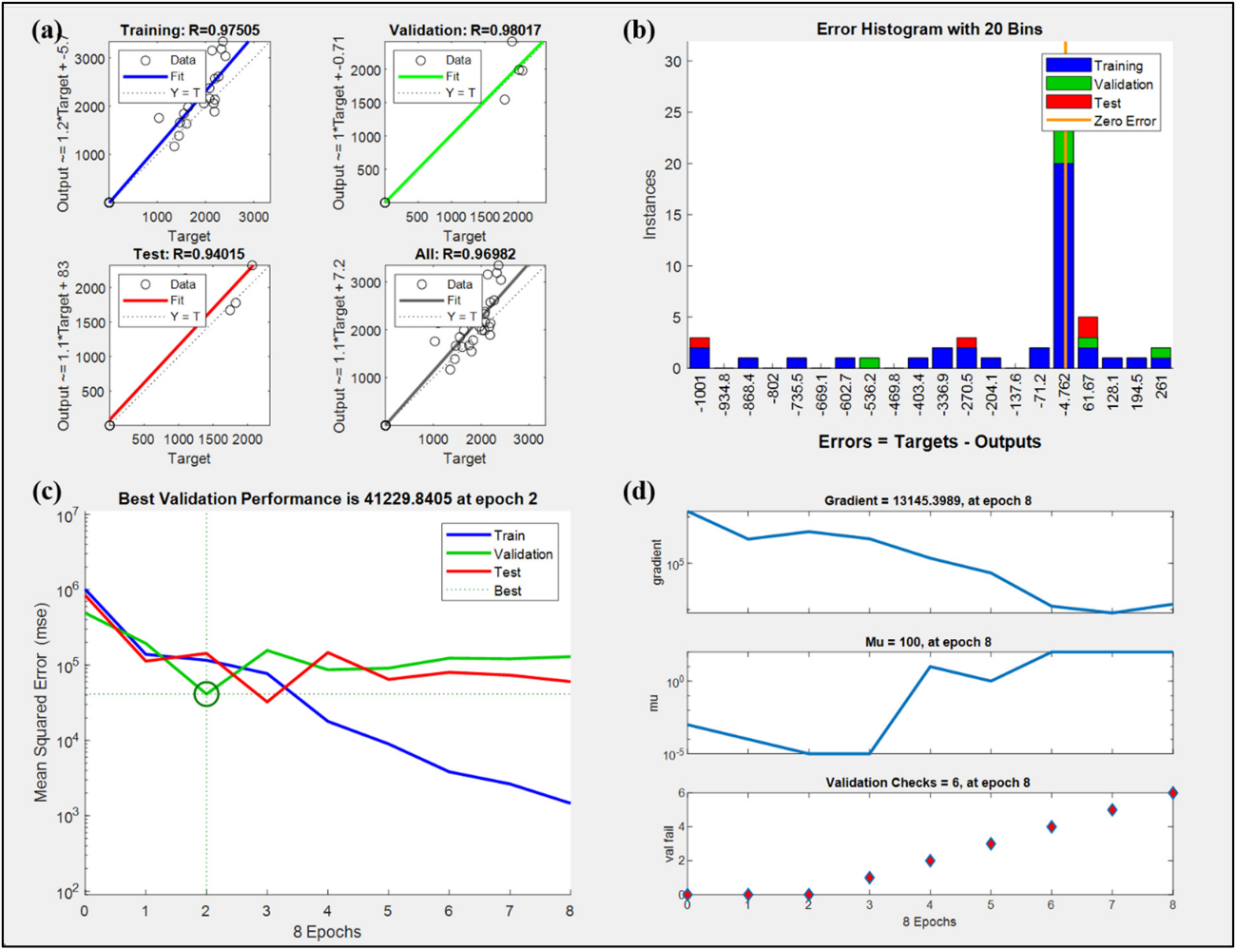
Levenberg-Marquardt backpropagation model for CRC and VMS of EN36C steel.

Figure 8 illustrates the Levenberg-Marquardt backpropagation model for CRC and VMS. Figure 8(a) shows the regression plots for the training, validation, test, and overall datasets, comparing the target values (actual) with the output values (predicted), along with the corresponding R-values, which represent the correlation between the predicted and actual values. The training plot illustrates the model's performance on the training dataset, represented by the blue line. The correlation coefficient (R = 0.97505) indicates a strong positive linear relationship between the predicted and actual values. However, the equation Output = 1.2 × Target−5.7 reveals a slight bias, as the slope deviates from the ideal value of 1, suggesting the predictions are consistently scaled slightly higher than the actual values. The validation plot shows the validation dataset, where the green line represents the model's predictions. The correlation coefficient (R = 0.98017) is higher than that of the training dataset, suggesting that the model generalizes well to unseen validation data. The equation Output = 1 × Target−0.71 shows a nearly perfect alignment with the ideal fit, as indicated by the dotted line (Y = TY = TY = T). Further, test plot evaluates the model's performance on the test dataset, represented by the red line. Here, the correlation coefficient (R = 0.94015) is slightly lower than those of the training and validation datasets, indicating some reduction in predictive accuracy. The equation Output = 1.1 × Target + 83 highlights a slight bias in the model's predictions for this dataset, as the intercept and slope deviate from the ideal values. Atlast, the overall plot combines all datasets (training, validation, and test) to evaluate the model's overall performance. The correlation coefficient (R = 0.96982) confirms that the model achieves strong predictive capability across all data points. The equation Output = 1.1 × Target + 7.2 indicates a small but consistent deviation from the ideal fit, suggesting that the model might benefit from further refinement to minimize bias and improve generalization.

Figure 8(b) illustrates the distribution of prediction errors from a machine learning model across the training, validation, and test datasets. Errors are calculated as the difference between the actual target values and the predicted outputs (Error = Target−Output). It was observed that majority of errors are clustered around zero error, suggesting that the model has performed well in minimizing error, particularly for the training data, which dominates the central bins. However, the error distribution exhibits some outliers, as indicated by the nonzero instances in bins farther from the zero line. These outliers are present in all three datasets, with test data and validation data showing higher dispersion compared to training data. This pattern may indicate that while the model performs well on the training set, there is a slight generalization issue for unseen data, leading to larger prediction errors in the validation and test datasets. Overall, the model demonstrates good overall predictive accuracy, as evidenced by the concentration of errors near zero, the presence of outliers and a broader distribution in the test and validation datasets, which suggests potential areas for improvement.

Figure 8(c) represents the best validation performance plot for the developed model over eight epochs, using mean squared error (MSE) as the performance metric. The training curve shows a steady decrease in the model's error with the increase in epochs, suggesting that the model is learning and fitting the training data effectively. Moreover, the validation curve initially follows a similar downward trend and reaches its minimum MSE value of 41229.8405 at epoch 2 marking the best validation performance during the training process. However, after epoch 2, the validation error begins to increase, indicating that the model is starting to overfit the training data. This means the model is learning patterns specific to the training data, which negatively impacts its performance on unseen validation data. Also, the test curve follows a trend similar to the validation curve. The test error decreases during the initial epochs and stabilizes, showing no significant improvement after the second epoch. Therefore, it was interpreted that the optimal performance of the model occurs at epoch 2, where the validation error is at its lowest. Beyond this point, the divergence between the training curve and the validation/test curves highlights overfitting, as the training error continues to decrease while the validation and test errors stabilize or increase.

Figure 8(d) shows the ANN performance during training over eight iterations (epochs), which contains subplots representing gradient, Mu and validation check, respectively. The gradient represents the rate of change of the loss function with respect to the model parameters. At epoch 8, the gradient is recorded as 13,145.3989, indicating the scale of adjustments being made to the model parameters. Over the course of training, the gradient steadily decreases, reflecting the model's convergence towards a local minimum of the loss function. This behaviour is typical for well-behaved optimization processes, as smaller gradients suggest that the model is approaching an optimal solution. The Mu plot illustrates the behavior of Mu, which is a parameter in the Levenberg-Marquardt optimization algorithm that governs the trade-off between the gradient descent and Gauss-Newton methods. At epoch 8, Mu reaches a value of 100. Initially, Mu starts very low, indicating a gradient descent-dominant approach. As training progresses, Mu increases, indicating a transition towards a Gauss-Newton method to fine-tune the parameters as the model nears convergence. The validation checks plot count the number of consecutive epochs where the validation performance fails to improve. At epoch 8, there are 6 validation checks, suggesting that the validation error has not improved for several epochs. This is often an indicator of overfitting or diminishing returns in training and typically signals that the training should be stopped. The increasing number of validations checks further supports the observation that the model has likely reached its optimal capacity and is no longer improving on unseen data.

### Mechanism of chip formation

**Figure 9.**
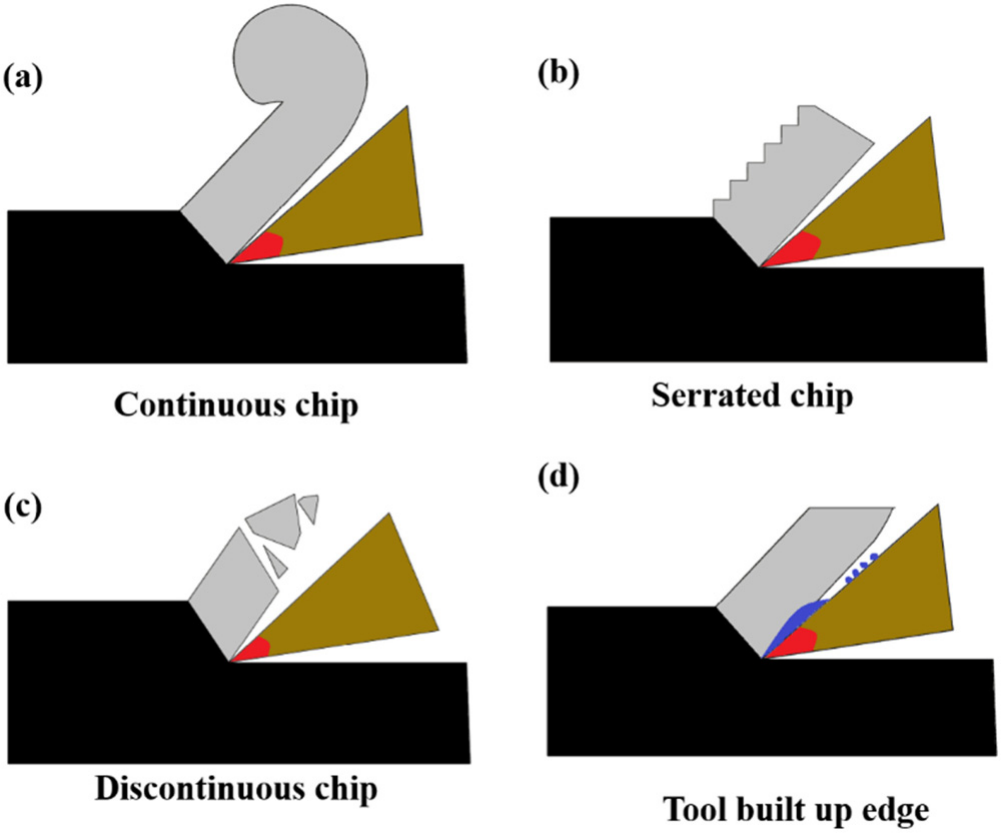
Schematic illustration of (a) Continuous chip, (b) Serrated chip, (c) Brittle chip, and (d) Built-up-edge (BUE) formation.

During the dry turning of steel, chip formation occurs due to intense plastic deformation in distinct zones influenced by cutting parameters such as speed, feed, and depth of cut (DOC). The process involves the primary shear zone, where material experiences extreme shear deformation to form the chip, the secondary shear zone, where friction between the chip and the rake face of the tool causes additional thermal deformation, and the tertiary shear zone, which occurs at the tool-workpiece interface. The type of chip formed depends significantly on the machining conditions. Figure 9 shows the schematic of the chip formation and built-up edge formation during the turning of metals. Figure 9(a) shows continuous chips formation under high cutting speeds and low DOC. These chips are characterized by smooth and uniform material flow, indicative of ductile deformation. Continuous chips result in improved surface finish and reduced tool wear, making them desirable for efficient machining. However, under moderate machining conditions, serrated chips are observed, as shown in Figure 9(b). These chips exhibit alternating zones of plastic deformation and brittle fracture due to cyclic strain hardening and thermal effects. Serrated chips represent a transition between ductile and brittle behavior, reflecting the influence of intermediate cutting speeds and feeds. Additionally, brittle chips are formed at lower cutting speeds, high feed rates, and high DOC, as illustrated in Figure 9(c). These chips are characterized by cracking and fragmentation, indicative of the material's brittle response. Brittle chips are less desirable as they may lead to poor surface quality and higher tool wear due to the abrupt nature of material removal. Another significant phenomenon is BUE formation, shown in Figure 9(d). BUE occurs when workpiece material adheres to the rake face of the tool due to high friction and pressure. The adhered layer undergoes plastic deformation and temporarily acts as a protective barrier, reducing tool wear. However, BUE is often unstable and detaches during cutting, exposing fresh tool material and potentially damaging the workpiece surface. BUE formation is more pronounced at lower speeds and higher feeds, where frictional and adhesive forces are predominant. The interplay of chip morphology and BUE formation significantly impacts machining outcomes, including surface quality, tool life, and machining efficiency.

#### Continuous chip

**Figure 10.**
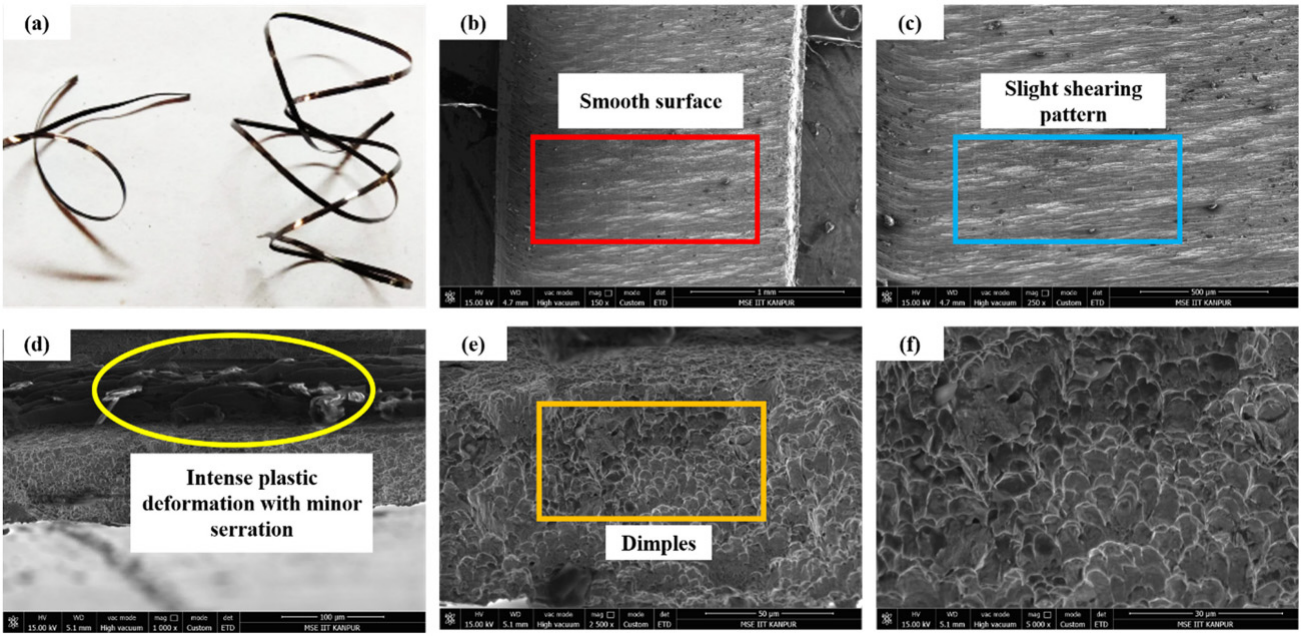
(a) Continuous chip formed under high speed and low feed/DOC; (b-f) SEM cross-sections showing smooth flow, shear patterns, and grain refinement due to varying machining stresses.

Figure 10 illustrates the continuous chip morphology of EN36C steel during dry turning, with Figure 10(a) showing the camera image of the smooth, ductile chip generated at high cutting speeds, low feed, and shallow depth of cut (DOC). The SEM cross-sectional region of the chip shown in Figure 10(b-f) provides detailed insights into the chip's microstructure. Figure 10(b) shows a smooth, aligned grain structure reflecting uniform material flow and lower VMS, typically showing ductile deformation. Figure 10(c) shows slight shearing patterns, indicating moderate stress concentrations under slightly elevated feed and DOC. Figure 10(d) reveals transitional features, with localized thermal effects and minor serrations due to increased mechanical stresses. At higher magnifications, as shown in Figures 10(e) and 10(f), the microstructure displays grain refinement and strain hardening caused by intense plastic deformation during chip formation. High cutting speeds minimize localized stress and promote continuous chip formation, whereas lower speeds or higher feed and DOC increase strain energy and VMS, leading to serrated or fractured chips. These observations highlight how machining parameters directly influence chip morphology and stress distribution during turning.

#### Serrated and semi-ductile chip

**Figure 11.**
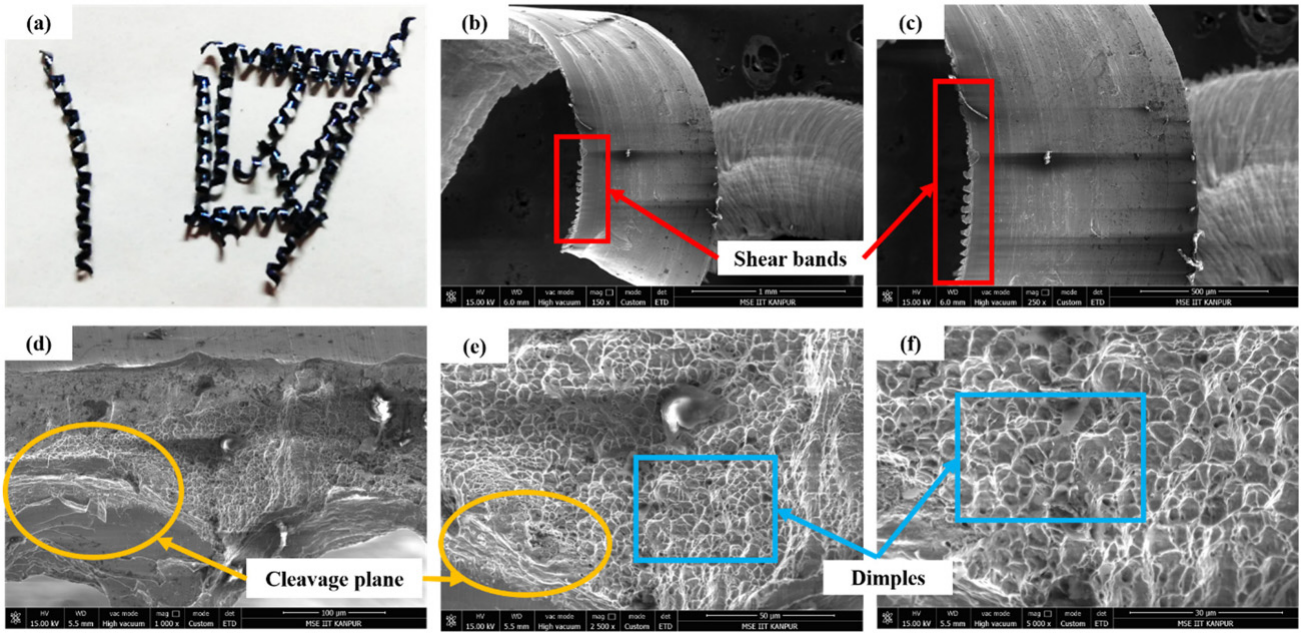
(a) Serrated semi-ductile chip formed under moderate speed and feed/DOC; (b-f) SEM images showing chip surface shear bands and cross-sectional features, indicating semi-ductile failure with a mix of ductile dimples and brittle cleavage planes.

Figure 11(a) shows the serrated curl chips, indicating periodic shear deformation due to semi-ductile failure. Figures 11(b) and 11(c) reveal the chip surface with distinct shear bands, caused by localized shear deformation under the influence of VMS, which governs material flow and fracture. Figures 11(d) and 11(f) highlight the chip cross-section, showing a mix of dimples (ductile features) and cleavage planes (brittle features), characteristic of semi-ductile failure. Moderate cutting speed increases the temperature, reducing the material's yield strength and enabling controlled serration without excessive thermal softening. The feed rate at moderate levels maintains chip segmentation by balancing stress and strain. Similarly, a moderate depth of cut (DOC) controls stress concentration, avoiding extreme brittle or ductile failures. These conditions result in balanced thermal and mechanical effects, evident from the shear bands and fracture patterns. The semi-ductile nature of chip formation demonstrates the interplay of cutting speed, feed rate, and DOC with the VMS distribution, enabling effective material removal.

#### Brittle and discontinuous chip

**Figure 12.**
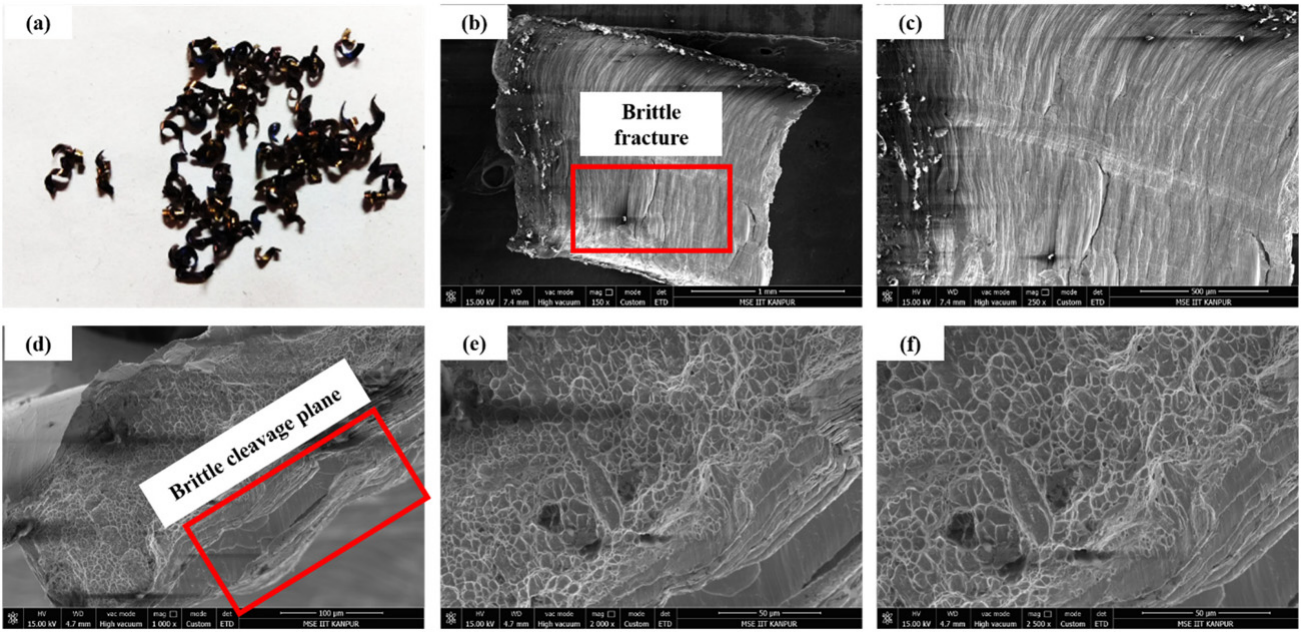
(a) Discontinuous chip formed under low speed and high feed/DOC; (b-f) SEM images showing surfaces and cross-sections dominated by brittle cleavage planes with limited ductile deformation.

Figure 12(a) shows the brittle chips characterized by fractured and fragmented shapes due to excessive stress concentrations. Figures 12(b) and 12(c) highlight the chip surface, revealing fewer or no shear bands, indicative of a predominance of brittle behavior. Figures 12(d) and 12(f) show the SEM of the chip cross-section, where brittle cleavage planes dominate, with minimal ductile dimples. At lower cutting speeds, reduced thermal softening results in higher material strength, promoting brittle fracture. High feed rates increase the cutting force, generating higher stresses that exceed the material's ductility limits. Similarly, high DOC amplifies stress concentration at the cutting edge, leading to catastrophic brittle failure. The VMS in this scenario exceeds the material's fracture strength, resulting in fragmented chip formation and limited plastic deformation.

### Comparative analysis with prior studies

The chip morphology observed in this EN36C study parallels behavior reported for other steels under dry turning. Continuous, ductile chips formed at high cutting speeds (100 m/min) and low feed in our tests, whereas low-speed/high-feed conditions produced fragmented brittle chips. Similar trends have been noted in another study where Cr–Mn austenitic steel yielded broken chips at low parameters but long tubular (continuous) chips at higher speed, feed, and depth.^32^ Kumar and Chakraborty^33^ reported lamellar shear-sliding chips under moderate feeds that developed cracks at higher speeds/feeds, which in turn relieved stress in the chip. These observations align with our SEM analysis, where higher speeds soften the workpiece material and promote ductile chip flow, resulting in smoother surfaces and reduced tool wear, whereas lower speeds and deeper cuts lead to brittle fracture and built-up edge formation. Also, in the current study, ANOVA attributed most of the variation in CRC and VMS to speed (52.0% and 35.0%, respectively), with depth having minimal effect. This deviation likely reflects EN36C's case-hardened structure; thermal softening at high speed has an outsized influence on chip thinning compared to geometric factors.

## Conclusion and future scope

This study focuses on predicting optimized parameters for the dry turning of EN36C steel to enhance machining efficiency. The output responses, CRC and VMS, were minimized using ANOVA and the Levenberg-Marquardt (LM) backpropagation ANN model. Results indicate that cutting speed (100 m/min) has the most significant impact on CRC and VMS, with feed rate (0.63 mm/rev) and depth of cut (1.0 mm) playing secondary roles. ANOVA attributed 52.04% of CRC variation and 35.04% of VMS variation to cutting speed, while the ANN model achieved 97% predictive accuracy. SEM analysis revealed a strong correlation between machining parameters, chip morphology, and material behavior. At high speeds, low feed, and low depth of cut, continuous chips were formed, characterized by smooth, aligned grain structures that reflected uniform material flow, lower VMS, and ductile deformation. Under moderate cutting conditions, semi-ductile serrated chips were observed, exhibiting mixed brittle and ductile features such as cleavage planes and dimples. At the lowest speeds with high feed and depth of cut, the morphology transitioned to brittle chips due to increased cutting forces and stress concentrations. High feed rates elevated cutting forces, exceeding the material's ductility limits, while high depth of cut amplified stress at the cutting edge, leading to catastrophic brittle failure. In this scenario, VMS surpassed the material's fracture strength, resulting in fragmented chips and limited plastic deformation. The findings of this study offer valuable insights for industry practitioners working with hard-to-machine alloys like EN36C. By identifying cutting speed as the dominant factor influencing both CRCand VMS, this research enables manufacturers to optimize dry turning parameters to enhance productivity while minimizing tool wear and material stress. The validated ANN model provides a powerful tool for predicting machining outcomes without the need for exhaustive trial-and-error experiments, reducing setup time and operational cost. These insights can support more efficient planning of machining operations for gear and shaft production, improve surface integrity, and extend tool life in dry machining environments.

While this study successfully models the CRC and VMS during the dry turning of EN36C steel, future research could expand on these findings by exploring a broader range of materials, including other case-hardened or heat-treated steels. Incorporating additional output parameters such as surface roughness, cutting forces, and tool wear would offer a more comprehensive understanding of machining behavior. Furthermore, applying hybrid predictive models such as combinations of artificial neural networks with genetic algorithms or fuzzy logic could enhance model accuracy and adaptability. Investigating the effects of alternative cooling methods like minimum quantity lubrication (MQL) or cryogenic cooling would also be valuable for assessing sustainable machining performance. Finally, integrating finite element simulations could provide deeper insights into chip formation mechanisms and stress distribution, complementing the experimental results and aiding in the development of more generalized machining optimization frameworks.
